# Injection Treatment of Neuritis with Hot Saline Solution

**Published:** 1916-12

**Authors:** 


### INJECTION TREATMENT OF NEURITIS WITH HOT SALINE SOLUTION.

Some years ago, injections of alcohol into the nerve tissue were recommended for intractable neuritis, for example in sciatica and in tic douloureux. In an effort to determine whether alcoholic injections into the nerve tissue are innocuous or not, Dr. Alfred Gordon found that these injections may give rise to decided degenerative changes. He has, therefore, abandoned the alcoholic injections in favor of saline solution of high temperature (*Ther. Gaz.*, June, 1916, p. 392), from which latter he has obtained highly satisfactory results. These injections were repeated at intervals varying from four days to two weeks.

While the time is too short to claim permanent cures, the relief experienced by his patients has been so decided and continuous, that the author recommends this method in the most emphatic manner. Saline solutions of high temperature give better and more lasting results than when the fluid is tepid or cold.—*Clinical Medicine.*

